# Perceptions of Health System Professionals on Integrating Fertility Care into Reproductive Health Policy in China

**DOI:** 10.3390/healthcare13050555

**Published:** 2025-03-04

**Authors:** Liu Zhang, Dongping Qiao

**Affiliations:** School of Government, Beijing Normal University, Beijing 100875, China; zhangliu@bnu1.org

**Keywords:** reproductive health policy, fertility care policy, health system, professionals, infertility, qualitative research, China

## Abstract

**Background**: Infertility is a neglected global public health issue, particularly in the Global South, where policy interventions and research remain limited. In China, rising public demand and declining birth rates have renewed interest in integrating fertility care into reproductive health policies, though operational challenges and systemic gaps persist. **Objectives**: This study aims to explore the perceptions of health system professionals regarding the opportunities and barriers to integrating fertility care into China’s reproductive health policy. **Methods**: This qualitative study involved 31 interviewees, including health system leaders (n = 5), health practitioners (n = 21), and civil society advocates (n = 5), from November 2023 to October 2024. The transcribed and anonymized data were thematically analyzed using MAXQDA version 2020, guided by the World Health Organization’s health system building blocks framework. **Results**: Interviewees reported that integrating fertility care has markedly improved service accessibility and quality, driven by strong governmental leadership. They identified opportunities for further progress through focused government initiatives, expanded public–private partnerships, and the adoption of international best practices, while also noting obstacles such as operational challenges, systemic policy gaps, uneven resource distribution, and persistent cultural stigma. **Conclusions**: The findings underscore the need for a robust national policy framework, sustainable funding mechanisms, and enhanced primary healthcare capabilities, along with cultural advocacy and awareness campaigns to reduce stigma and foster equitable access.

## 1. Introduction

Infertility is a globally significant yet often neglected public health issue. The World Health Organization (WHO) defines infertility as a disease of the male or female reproductive system characterized by the inability to achieve pregnancy after 12 months or more of regular unprotected sexual intercourse. It is estimated that approximately one in six individuals worldwide experiences infertility during their lifetime, affecting approximately 17.5% of the adult population [1]. This condition imposes profound emotional and economic pressures on individuals and families while also posing significant public health challenges [2]. The 1994 International Conference on Population and Development (ICPD) first highlighted the importance of infertility prevention and management as an integral component of sexual and reproductive health rights (SRHR) [3]. Since then, global health policies and strategies have underscored the need for fertility care. However, infertility remains a low priority in public health policies, particularly in resource-constrained settings in the Global South [2].

In recent years, global attention to infertility has increased, with some countries expanding investments in research, policies, and practices [4,5]. For instance, countries such as India, Iran, and several African nations face challenges including limited resources, socioeconomic disparities, and entrenched cultural stigmas, all of which impede the integration of fertility care into national health systems [6,7,8]. In The Gambia, West Africa, infertility is being recognized as a significant public health burden, prompting health authorities to incorporate specific measures into the national reproductive health strategy [9]. Addressing these challenges requires affordable, context-specific fertility care services supported by strong political commitment and community engagement [10]. These cross-national comparisons underscore the need to better understand both the shared barriers and context-specific facilitators to develop robust, equitable, and sustainable fertility care policies.

In many national health agendas, fertility care is overshadowed by more established public health priorities, such as maternal health, contraception, and HIV/AIDS [11]. Although assisted reproductive technologies (ART) represent a key component of infertility treatment, their prohibitive cost limits accessibility within many national health systems, while other fertility care interventions remain comparatively less expensive. Moreover, even when fertility care is incorporated into health policies, its implementation frequently exacerbates inequities, with disparities emerging along rural–urban, socioeconomic, educational, and gender lines [12]. Translating policy intentions into concrete actions requires robust engagement across both public and private sectors, coupled with a comprehensive understanding of the power dynamics and perspectives of policymakers and health practitioners [13].

China faces a particularly acute challenge, with a declining birth rate compounded by rising infertility rates. The national birth rate decreased from 23.33 per thousand in 1987 to 6.77 per thousand in 2024 [14]. This decline reflects a confluence of factors, including reduced fertility intentions driven by socioeconomic pressures and an increasing prevalence of infertility [1,15]. Over the past two decades, the national infertility rate has risen from 11.9% in 2007 to 17.6% in 2020, affecting an estimated 33 million couples [16].

Historically, infertility was overlooked in China due to the one-child policy implemented in the early 1980s to curb overpopulation [17]. Although this policy was applied differently in urban and rural areas, it contributed to the neglect of reproductive health challenges. With the gradual relaxation of the policy starting in 2013 and the subsequent implementation of the universal two-child policy in 2015, expectations for birth rate rebound were not fully realized. Although live births initially increased from 16.55 million in 2015 to 17.86 million in 2016, the numbers steadily declined in the following years [17]. This trend is partly due to an increase in advanced maternal age, as more women over 35 delay childbearing, thereby accelerating infertility as a result of the natural decline in oocyte quality [18]. Additionally, environmental exposures, chromosomal abnormalities, lifestyle factors, and other unexplained variables further exacerbate this challenge [16].

Recognizing infertility as a significant public health burden, the National Health Commission of the People’s Republic of China (NHC) has initiated efforts to integrate fertility care into national health policies, including the incorporation of ART into medical insurance schemes across all 31 provinces. However, research on the opportunities and barriers to integrating fertility care into reproductive health policies in China remains limited. Understanding these dynamics is crucial not only for addressing China’s unique demographic and public health challenges but also for informing similar initiatives in other Global South countries.

This study applies the WHO health system building block framework to analyze the structural barriers and enablers of integrating fertility care into China’s reproductive health policies [19]. It aims to explore the perceptions of health system professionals regarding opportunities and challenges in integrating fertility care into these policies, thereby providing actionable insights to inform public health practices in China and other developing countries facing similar challenges.

## 2. Methods and Materials

### 2.1. Study Design and Setting

This qualitative study was conducted between November 2023 and October 2024 in two provinces of China, namely Beijing and Hunan. These regions were selected for their historical and policy significance. Beijing, recognized as the birthplace of China’s first in vitro fertilization (IVF) baby, represents the origin of assisted reproductive technologies (ART) in the country. Hunan Province is notable for implementing the nation’s first embryo donation IVF case and establishing China’s first sperm bank, thereby marking significant milestones in policy and technological innovation. This selection reflects the diversity of clinical practices and policy approaches across regions. This study focused on professionals involved in reproductive health, including government officials, healthcare providers (doctors and nurses), and organizational staff engaged in fertility care advocacy and awareness-raising activities. Patients and the general public were not included in this article.

### 2.2. Sampling and Recruitment of Participants

Participants were recruited using purposeful sampling, targeting individuals with direct experience in health policy formulation and implementation to elicit in-depth insights into reproductive health policies and practices. Additionally, snowball sampling was employed to capture diverse perspectives and participant backgrounds. Participants were categorized into three groups based on their roles within the health system:

(i) Health System Leadership: Policymakers and policy implementers responsible for strategic decision making, including representatives from health system officials at the central and regional levels. The National Health Commission of the People’s Republic of China (NHC), in the past known as the Ministry of Health (MoH), is a cabinet-level executive department of the State Council of the People’s Republic of China responsible for formulating national health policies.
(ii) Health Practitioners: Professionals directly involved in service delivery, subdivided into:
  - Tertiary Care Providers: Specialized doctors and nurses in public hospital settings, typically with advanced training and experience in reproductive health services.
  - Primary Care Providers: General doctors and nurses in primary medical facilities, often serving as the first point of contact for patients seeking fertility care.
(iii) Civil Society Advocates: Representatives from civil society organizations engaged in reproductive health advocacy and awareness-raising activities within both rural and urban communities. These individuals often have backgrounds in public health, social work, or related fields and play a crucial role in community education and support.

Recruitment support for health practitioners was provided by the LY Foundation (https://www.chnjkfp.org/, accessed on 18 January 2025), a non-profit organization that advocates for low-cost IVF treatments. As the only non-profit organization in China conducting such initiatives, the Foundation played a critical role in facilitating participant recruitment. In the first stage, key informants were selected with the support of the Foundation. In the second stage, the sampling process followed a purposive sampling framework, considering variables such as occupational category and professional level. Additionally, two focus groups were conducted—one with LY Foundation staff and one with primary care providers—to provide contextual insights into patient experiences. Data collection continued until no new themes emerged, and an acceptable interpretative framework was established.

### 2.3. Data Analysis

Interview transcripts were transcribed verbatim, anonymized, and analyzed using MAXQDA 2020, a qualitative data analysis software. The analysis was guided by the WHO health systems building blocks framework, which comprises six components: (i) leadership and governance; (ii) health information system; (iii) health financing; (iv) service delivery; (v) medicines and technologies; (vi) health workforce [19].

Thematic analysis was conducted to identify key opportunities and barriers in the implementation of fertility care policies. The findings were subsequently used to develop evidence-based recommendations for improving policy and practice in China and other developing countries.

### 2.4. Ethic Approval

The research proposal received ethical approval from the Doctoral Research Programme & Ethics Review of the School of Social Development and Public Policy (SSDPP) at Beijing Normal University on 15 September 2023 (No. SSDPP202131240002). All participants of this study received an information sheet and signed informed consent forms prior to participation. Participants were informed of this study’s objectives, the voluntary nature of their involvement, and the research team’s commitment to maintaining anonymity and confidentiality during data processing. Data were managed in accordance with Chinese national legislation on personal data protection.

## 3. Results

A total of 31 key informants were recruited, including 5 representatives of health system leadership, 21 health practitioners, and 5 civil society advocates. The participants comprised 13 males and 18 females, with the majority being doctors (56.1%). Regarding professional levels, most held primary-level (35.5%) or intermediate-level (32.3%) positions. The age distribution was as follows: 36–45 years (38.7%) and 46–55 years (29%). In terms of education, some primary care providers had completed only high school (25.8%). Geographically, participants were from both rural (25.8%) and urban (74.2%) areas. Table 1 provides an overview of the participants’ demographic and professional characteristics.

### 3.1. Leadership and Governance

The inclusion of fertility care in China’s national reproductive health policy has significantly improved the quality and accessibility of public services, underscoring the government’s central role in addressing this pressing public health challenge. Interviewees universally recognized the transformation of government health department functions and the introduction of supportive policies as key drivers in elevating infertility to a national public health priority. Notably, Chinese policy documents explicitly recognize infertility as a reproductive health burden. This strategic policy framing has been instrumental in building consensus and advancing public recognition of infertility as a legitimate public health concern.

Although implementation continues to evolve, several opportunities have emerged to further strengthen policy success. Some policymakers observed that while addressing infertility is crucial for comprehensive reproductive health, the primary focus on increasing fertility rates may sometimes overshadow these efforts. One policymaker stated, *“The infertility issue deserves attention, but its role in reversing the fertility rate may be limited” (Policymaker, male)*. In addition, interviewees expressed diverse views on the classification of infertility, with one noting, *“Questions such as Is infertility a disease? Is it the most important disease?” (Policymaker, female)*. These varying perspectives highlight the need for continued dialogue to align understandings and reinforce the policy’s achievements. Enhanced regional coordination was also identified as a promising avenue to further support effective policy implementation at the local level.

Opportunities to expand collaboration among health system professionals have also emerged. While current cooperation among public health sectors, private institutions, social organizations, and commercial insurance entities has advanced policy implementation, further integration could enhance outcomes. As one civil society advocate remarked, *“The voices of the patient group are rarely heard in policy making” (Civil Society Advocate, male)*, suggesting that incorporating grassroots perspectives could further enrich policy development. Primary care providers also suggested that integrating local insights into the policy formulation process would add valuable depth to the national strategy. Expanding multi-stakeholder participation and fostering inclusive, transparent decision-making mechanisms were seen as key facilitators in strengthening the overall policy framework.

Finally, there is significant potential to broaden public education and advocacy efforts, particularly in rural areas. Although cultural taboos and stigma have historically limited the dissemination of infertility-related knowledge—one doctor explained, *“When we give lectures in remote rural areas, we can only use the banner of gynecological diseases or maternal and baby healthcare” (Tertiary Care Provider, female)*—interviewees emphasized the importance of comprehensive, nationwide public education campaigns to raise awareness and foster acceptance.

### 3.2. Health Information System

An effective health information system is indispensable for optimizing policies and allocating resources. China’s current system primarily focuses on clinical treatment data and is evolving to incorporate broader dimensions. For instance, the quality monitoring system for ART, overseen by the NHC, provides nationwide data but centers primarily on technical operational metrics within medical institutions. Future integration of additional dimensions, such as patients’ financial burdens, treatment demands, and social support, is under consideration.

While all 31 provinces in China included ART treatments under medical insurance by 2024 [20], there is an opportunity to further enhance evaluation of policy implementation outcomes through the development of a unified monitoring mechanism. One policymaker observed that current approaches are fragmented at the national level, suggesting that a more coordinated management framework could further optimize policy outcomes.

Data collection methods vary significantly between medical institutions and social organizations. Physicians typically report patient information through hospital systems, which relies on a passive approach to patient engagement. In contrast, community organizations employ proactive methods, such as screening for potential patients. One doctor noted, *“Community maternal health directors observe families’ behaviors during folic acid distribution or prenatal consultations to identify potential fertility issues before referring them to us” (Tertiary Care Provider, female)*. This proactive approach by community organizations serves as a promising facilitator for identifying and addressing fertility care needs.

Although comprehensive data collection at the community level remains limited, primary care providers in rural communities often have valuable insights about couples facing infertility. One male primary care provider explained, *“In rural communities, primary care doctors often know which married couples have not been able to conceive; however, infertility is considered a private matter, making it difficult to address proactively” (Primary Care Provider, male)*. In contrast, female primary care provider reported taking a more proactive stance when they had a closer relationship with the patient: *“I sometimes take the initiative to ask, especially if I have a close relationship with the patient” (Primary Care Provider, female)*.

Civil society advocates underscored the critical role of community engagement in data collection. One advocate stated, “Community-based organizations are often the first to identify individuals facing fertility challenges.” (Civil Society Advocate, male) emphasizing the potential for these organizations to contribute more systematically to national health data systems. They stressed that integrating community-generated insights into national health data systems could create a more holistic and comprehensive understanding of infertility challenges and further enhance policy success. Integrating data-sharing mechanisms among health departments, social organizations, and medical institutions represents a promising avenue for enhancing decision making and policy effectiveness.

### 3.3. Health Financing

Participants mentioned that families often opt for ART after trying medications and surgery for organic causes. Although only approximately 10–20% of infertile couples need to undergo ART [21], this treatment represents a significant portion of the overall cost burden compared to other interventions, such as artificial insemination, clomiphene, surgery, and observation, which together account for 62% of all treatments yet only 10–20% of total treatment costs [22,23].

Primary care providers expressed positive views on the fertility care policy, noting that the reduction in treatment costs has alleviated the financial burden on families. One doctor stated, *“New policy has significantly reduced the financial burden on families, making it more accessible to those in need” (Primary Care Provider, male)*. They also suggested that further expanding reimbursement for rural residents could enhance equitable access to fertility care.

Civil society advocates similarly emphasized the value of universal economic support mechanisms in ensuring equitable access. They agreed that the integration of ART into medical insurance has reduced treatment costs and alleviated financial burdens for many families, while also identifying further opportunities to enhance reimbursement rates and establish broader economic support mechanisms.

While ART treatment is now covered by medical insurance nationwide, current financing models prioritize basic and widely used treatment services. One policymaker noted, *“It is impossible to cover all projects or provide high-level protection at once” (Policymaker, female)*. This pragmatic approach has successfully reduced treatment costs, with promising opportunities for future expansion of coverage as financing capacities grow.

Diversified financing mechanisms are viewed as a promising avenue to further enhance the sustainability of fertility care. Interviewees advocated for the establishment of special funds and greater involvement of social organizations and commercial entities. Public welfare projects in certain regions have successfully reduced treatment costs for low-income families, making care more accessible. One practitioner remarked, *“Philanthropic projects have reduced treatment costs, enabling many low-income families to afford care and encouraging more patients to seek treatment” (Tertiary Care Provider, female)*.

China is also addressing the challenges associated with an aging society and evolving financial dynamics. Forecasts research suggest that, under the current healthcare system, the balance of income and expenditure of China’s medical insurance fund may face challenges, with projections indicating an annual shortfall by 2026 and a cumulative shortfall by 2034 [24]. Despite these forecasts, interviewees both from policymakers and policy implementers emphasized that affordability remains the cornerstone of the medical insurance system. They maintained that, as long as existing Medicare claims are sustained, robust support for the examination and treatment of infertility should continue nationwide. It is widely recognized that medical insurance should favor vulnerable groups, and efforts to reduce or control out-of-pocket costs for economically disadvantaged patients are recommended as a means to further highlight the significant role of medical insurance in fertility care.

Future policy design should also consider addressing ancillary patient costs, such as work disruptions, psychological stress, and long-term care requirements. For example, many women undergoing infertility treatment face employment challenges; one practitioner noted, *“Many female patients must choose between resigning from their jobs or abandoning treatment altogether” (Tertiary Care Provider, female)*. Recognizing and mitigating these hidden costs will further enhance the overall success of fertility care policies.

### 3.4. Service Delivery

The integration of infertility diagnosis and treatment into China’s national reproductive health service framework marks substantial progress. While resource distribution shows variability between urban and rural areas, initiatives are emerging to expand access. As of 2024, China had 622 certified ART medical institutions, predominantly concentrated in urban centers. Pilot projects in some regions have implemented effective referral mechanisms between primary care centers and higher-level hospitals. For instance, township health centers collaborate with hospitals to identify potential patients, thereby improving access to care. One practitioner explained, *“We keep in touch with doctors in township clinics and teach them how to assess whether a patient is likely to be infertile. If they suspect infertility, they refer the patient to us” (Tertiary Care Provider, female)*. This model demonstrates promise for further expansion in under-resourced areas.

Community-based primary healthcare providers play a pivotal role in delivering psychological counseling and case management for patients undergoing fertility treatments. Often serving as the first point of contact, they offer essential emotional support and guidance throughout the treatment process. One primary care provider noted, *“I spend a significant amount of time on psychological counseling for patients, helping them cope with emotional challenges” (Primary Care Provider, female)*. Civil society organizations have been instrumental in advocating for the inclusion of psychological services and comprehensive case management in fertility care. They emphasized a holistic approach that addresses both the physical and emotional dimensions of infertility, thereby enhancing patient well-being and treatment outcomes.

### 3.5. Medicines and Technologies

Reducing treatment costs is critical to equitable access to fertility care. Interviewees reported that ART expenses are primarily divided among surgical procedures (50%), medications (30%), and diagnostic tests (20%). While the integration of ART into medical insurance has already alleviated a portion of the financial burden for eligible families, high-cost medications and essential diagnostic procedures—both excluded from current reimbursement frameworks—remain financially prohibitive for marginalized populations.

Non-medical approaches, such as traditional Chinese medicine and acupuncture, are frequently utilized in fertility care and align with patient preferences and cultural practices. Expanding the policy scope to incorporate these alternatives could further benefit patients seeking holistic treatment options. While infertility care requires specialized equipment and a range of supplies, not all treatment protocols necessitate expensive drug regimens. The LY Foundation is actively working to reduce the overall cost of infertility treatment, thereby enhancing accessibility for a broader population. One tertiary care provider observed, *“Enhancing the capabilities of local clinics is essential for offering comprehensive fertility care to the community” (Tertiary Care Provider, female)*.

### 3.6. Health Workforce

High-quality fertility care relies on a robust and well-trained medical workforce. Enhancing reproductive medicine training within China’s medical education system is a key area of ongoing development. Many practitioners acquire expertise through on-the-job training, and capacity-building initiatives are underway to ensure that primary healthcare facilities have access to skilled professionals. These initiatives are essential to promoting equity in service delivery across both urban and rural regions.

All of the interviewees emphasized optimizing medical curricula, expanding professional training programs, and improving certification mechanisms as strategies to further strengthen the workforce. One policy implementer noted, *“Enhancing primary care physician training is the key to improving the coverage of services” (Policy Implementer, male)*. Primary care providers and civil society advocates alike highlighted the critical need to integrate fertility care training into medical education. One civil society advocate stated, *“Training healthcare professionals in fertility care is essential for empowering communities and reducing stigma” (Civil Society Advocate, male)*. A community-based doctor mentioned, *“The recent policy changes have provided us with more resources and support to assist couples struggling with infertility.” (Primary Care Provider, female)* These perspectives underscore the importance of continued investment in workforce development to ensure equitable access to fertility services across all regions.

**Figure 1:**
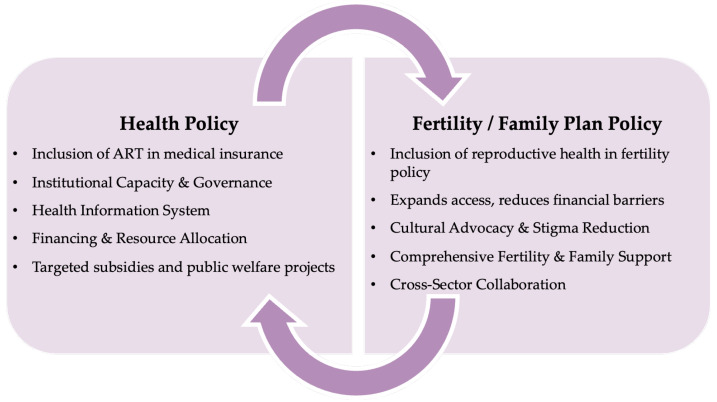
Conceptual framework: enablers to fertility care policy making.

Building on the findings presented in the results section, we developed a conceptual framework to guide fertility care policy making in China. This framework is organized into two primary dimensions— health policy and fertility/family planning policy—encompassing key enablers. These categories collectively capture the factors influencing the design, implementation, and sustainability of fertility care policies. Figure 1 provides an overview of these enablers, offering policymakers and professionals a structured approach to assessing and addressing the determinants of effective fertility care policy making.

## 4. Discussion

This study examined the facilitators and challenges of integrating fertility care into China’s reproductive health policy, as perceived by health system professionals. The findings demonstrate that this integration has benefited from strong governmental commitment, targeted policy directives, and innovative service delivery models. Health system professionals emphasized that clear policy measures, such as the recognition of infertility as a reproductive burden and the inclusion of ART in the national medical insurance system, have been decisive in transforming infertility from a traditionally neglected condition into a public health priority. In contrast to earlier studies that portrayed infertility as a low-priority issue due to its non-life-threatening nature [25], our findings highlight how shifts in population policies have elevated its status in China. Table 2 presents an overview of the key opportunities, barriers, and policy recommendations across the six health system components to provide a comprehensive framework to guide future policy development.

### 4.1. Infertility and ART in China

Infertility has historically been overlooked in China, partly due to the long-standing one-child policy [17]. Over the past two decades, infertility prevalence in China has risen sharply, with ART emerging as one of the most effective treatments [26]. China’s development of ART, particularly the birth of the first domestic test-tube baby in 1988, represents a significant milestone in the country’s reproductive health advancements. By 2024, the integration of ART into the medical insurance system marked a policy turning point, broadening access to these technologies and establishing infertility as a national public health priority. As noted in this study, all participants expressed strong support for the government’s transition from a focus on population control to prioritizing fertility care, which has significantly shifted the policy landscape. This transition has been instrumental in advancing fertility care.

However, resource distribution remains uneven, rural and remote areas continue to face substantial challenges. Interviewees in this study stated that efforts to enhance accessibility in underserved regions are critical to addressing these challenges. While urban centers, particularly in the eastern provinces, have robust ART services, rural and remote areas continue to face significant disparities in access [27]. These regional differences are compounded by financial and logistical barriers that place ART out of reach for many infertile patients in less developed areas [23]. This reflects a broader global trend where high-income countries have been more successful in subsidizing ART costs and integrating them into universal healthcare systems [25], whereas developing nations, including China, continue to confront challenges in expanding coverage. Nevertheless, the inclusion of ART in China’s national insurance scheme is a promising step forward, and efforts to enhance accessibility in underserved regions are critical for reducing these disparities.

### 4.2. Successes of Fertility Care Policies in China

Global concerns about overpopulation have historically overshadowed the importance of infertility treatment in policy priorities. However, there is growing recognition that reproductive health services should prioritize equity and social justice, ensuring that all individuals have access to the care they need to build a family. As reflected in this study, participants underscored the importance of continued dialogue and multi-stakeholder collaboration to strengthen policy success. Fertility care, which encompasses not only medical interventions but also fertility awareness campaigns and family-building support, plays a critical role in addressing low fertility rates and maintaining population balance [28]. The United Nations recognizes infertility as a component of sexual and reproductive health and rights, advocating for comprehensive approaches that ensure access to fertility care [29].

China’s population development strategy is transitioning from an emphasis on survival to an emphasis on thriving [17]. The overarching goal has shifted from reducing maternal and child mortality to achieving high-quality universal health coverage—with a continuing emphasis on access, equity, and quality, as well as nurturing a supportive environment for health and health services. As noted in this study, participants felt strongly that the government’s policy shift from focusing on population control to prioritizing fertility care has led to substantial changes, signaling a fundamental policy shift that has significantly advanced fertility care. Despite these advances, this article also pointed out that gaps in policy accessibility and public acceptance still remain, which need to be addressed for greater impact.

As a result of the one-child policy, China gradually entered a stage of low births, low deaths, and low natural growth. The government recognized that change was necessary. In 2011, the government began to relax restrictions on the one-child policy, allowing couples with a one-child status to have two children. In 2013, further relaxation permitted couples in which one spouse had a one-child status to have two children. However, these policies have had limited effects on increasing the birth rate. There is now a need for pronatalist policies, statutory maternity payments, and the provision of more affordable childcare and health care services [17]. This study also underscore that while ART policies have made substantial progress, there remain significant challenges in ensuring that fertility care is universally accessible, particularly in underserved regions.

China’s inclusion of ART in its medical insurance system marks a significant policy shift that has elevated infertility to a public health priority. However, despite these advancements, gaps remain in policy accessibility and public acceptance, which must be addressed. Compared to high-income countries where ART is more widely subsidized, China’s current approach is still in its early stages. As highlighted in this article, ART services are increasingly covered under national insurance, but regional disparities persist, with rural areas lagging behind urban centers in access to ART. For example, over 80% of European countries provide partial or full subsidies for ART [30], whereas many developing countries lag behind in offering such coverage. Our findings emphasize that these disparities continue to present significant challenges, and further efforts are necessary to improve policy accessibility, particularly in rural and underserved areas.

### 4.3. Fiscal Sustainability

Studies indicate that for every 1% increase in out-of-pocket payments as a share of total health expenditure, the incidence of catastrophic household spending rises by 2.2%; reducing out-of-pocket payments to below 15% can significantly diminish financial hardship [31]. Achieving universal health coverage requires addressing the financial challenges associated with infertility treatment. Primary care providers and civil society advocates in this study noted that reduced treatment expenses have eased family burdens. Infertility treatments, particularly ART, are costly. Although ART is now covered by medical insurance, current policies reimburse only partial surgical expenses. This leaves patients with significant out-of-pocket costs for medications, diagnostics, and other treatments. The high financial burden associated with ART is a challenge not only in China but also globally. Our findings highlighted that diversified financing mechanisms, such as targeted subsidies and the involvement of commercial insurance entities, are key to reducing this financial strain, which is consistent with previous studies [27,32].

International comparisons underscore the importance of diversified financing mechanisms. As noted in this study, interviewees advocated for the establishment of special funds and the greater involvement of social organizations and commercial entities to improve the sustainability of fertility care. In Finland, for example, national health insurance covered 60% of doctors’ fees and part of examination costs, and 50% of drug costs were reimbursed by the Social Insurance Institution [33]. Reimbursement for infertility treatment has also become a state-mandated insurance coverage in the USA [34]. In any country, the total cost of IVF did not exceed 0.25% of healthcare expenditure [35]. This findings point to the importance of moving towards specialized fertility funds or tax incentives for private sector participation to provide long-term financial support and reduce fiscal pressure on the healthcare system.

### 4.4. Addressing Fertility Inequality

Infertility is not only a medical issue but also a social equity issue, particularly in terms of gender equality. Primary care providers and civil society advocates in this study stressed the need for both financial and emotional support, as women disproportionately bear infertility’s psychosocial burden. In many cultures, including China, women bear the primary psychological and social burden of infertility, even when the cause is not biological [36]. Participants emphasized that culturally sensitive services and community-based emotional support are vital to improving outcomes and reducing stigma, aligning with prior research [29]. As highlighted in this article, rural primary care providers possess critical insights into infertility challenges, yet the topic remains stigmatized. As one doctor noted, infertility is perceived as a private concern, complicating proactive intervention. These findings underscore the need for comprehensive, inclusive fertility care policies that address both financial and emotional support, especially in underserved and rural areas.

China’s fertility care policies must account for the diverse needs of its population, particularly in rural areas where access to fertility services is limited. As noted in this article, primary care providers in rural communities often have valuable insights about couples facing infertility, yet addressing infertility proactively remains a challenge. Policy optimization should focus on increased investment in primary healthcare, improved medical education, and incentives to attract skilled professionals to underserved regions. These efforts can help reduce regional disparities and promote more equitable access to fertility care nationwide, as highlighted in this study.

### 4.5. Research Limitations

This study has several limitations. Although the qualitative design provided in-depth insights, it may not fully capture national trends, and the sample size may limit the generalizability of the findings. The focus on health system professionals excluded patients and the broader public, limiting the diversity of perspectives. This limitation arose from this study’s specific focus on policy and healthcare service delivery, as well as logistical constraints in directly reaching patients. Future studies should adopt mixed methods to include a wider range of stakeholders and explore the experiences of male patients and rural residents in greater depth. Additionally, the sensitivity of infertility may have restricted data collection, indicating the need for culturally adaptive research strategies in future studies.

While prior research has underscored the importance of including fertility care in public health agendas [1,10,11,13], this study contributes to the growing body of literature on fertility care policy by exploring implementation pathways in a middle-income country context. Future research should investigate collaborative stakeholder models and innovative financing strategies to enhance policy universality and public acceptance, ultimately ensuring that fertility care becomes universally accessible.

## 5. Conclusions

This study highlights the pivotal role of national policy in integrating fertility care into China’s reproductive health agenda. Our findings demonstrate that the inclusion of fertility care within the public health and family planning policy framework has significantly improved service accessibility and quality (Figure 1). The success of these policies is attributed to strategic government involvement and a coordinated multi-stakeholder financing model, which includes government, commercial insurance, philanthropic funding, and community support. While substantial progress has been made, further improvement is needed, including expanding public-private partnerships, enhancing regional access to services, and addressing the cultural stigma surrounding infertility. Given the global nature of fertility challenges, more research on fertility care policies in the Global South is essential to inform more equitable and inclusive reproductive health policies worldwide.

## Figures and Tables

**Table 1:** Key Characteristics of the Study Participants.

Characteristics	Male	Female	Total	
	(n = 13)	(n = 18)	(n = 31)	%
Profession				
Health System Leadership	3	2	5	16.1
- Policymaker	1	1	2	6.5
- Policy Implementer	2	1	3	9.7
Health Practitioners	7	14	21	67.7
- Tertiary Care Providers	2	9	11	35.5
- Primary Care Providers	5	5	10	32.3
Civil Society Advocates	3	2	5	16.1
Occupational category				
Government Official	3	2	5	16.1
Doctors	7	9	16	51.6
Nurses	0	5	5	16.1
Organization Staffs	3	2	5	16.1
Professional level				
Senior	2	3	5	16.1
Intermediate	3	7	10	32.3
Primary	5	6	11	35.5
N/A *	3	2	5	16.1
Age				
18–25	0	2	2	6.5
26–35	2	1	3	9.7
36–45	4	8	12	38.7
46–55	3	6	9	29.0
56+	4	1	5	16.1
Education				
doctor	3	2	5	16.1
master	2	6	8	25.8
bachelor	4	6	10	32.3
high school	4	4	8	25.8
Geography				
rural	5	3	8	25.8
urban	8	15	23	74.2

* Professional level is not available for civil society advocates.

**Table 2:** Opportunities, Barriers, and Recommendations for Integrating Fertility Care into China’s Reproductive Health Policy.

Six Components	National Policy Language	Opportunities	Barriers	Recommendations
Leadership and Governance	“Government is responsibility for reducing the costs associated with fertility, enhancing fertility care policies, and improving the maternity insurance system to alleviate the financial burden.” a	Explicit policy framing; Multi-stakeholder participation; Regional coordination.	Fertility rate focus; Divergent views.	Enhance stakeholder collaboration; Inclusive decision making; Strengthen regional coordination.
Health Information System	“Improve the quality control network, and strengthen service monitoring and the health information system.” b	National infertility database; Integrated data-sharing.	Fragmented data collection.	Unified monitoring; Enhance data integration.
Health Financing	“Taking into account factors such as the financial sustainability of medical insurance funds, efforts should be made to gradually include … and ART treatment services.” b	Nationwide ART coverage; Philanthropic cost reduction; Reimbursement expansion.	Partial reimbursement; Hidden out-of-pocket costs; Underused commercial insurance.	Tiered reimbursement; Targeted subsidies; Diversified financing.
Service Delivery	“Enhancing the accessibility of services provided by healthcare institutions.” c“Exploring effective models of integrated Traditional Chinese and Western medicine.” d	Successful pilot projects; Effective referral mechanisms; Expansion potential.	Limited primary care capacity; Inadequate referral systems.	Enhance primary care; Strengthen referral networks.
Medicines and Technologies	“Services including health education, psychological counseling, traditional Chinese medicine, pharmacotherapy, surgical treatments, and ART.” c	Expand insurance for meds/diagnostics.	High out-of-pocket costs.	Broaden insurance scope; Lower patient costs.
Health Workforce	“specialized healthcare professionals ensuring safe and effective fertility care.” e	Expand training; Increase capacity in underserved areas.	Shortage of skilled professionals in rural areas	Enhance training; Optimize curricula; Improve certification.

a. The Central Committee of the Communist Party of China and the State Council. “Decision on Optimizing Birth Policy to Promote Long-term Balanced Population Development”, released on 26 June 2021. b. NHC, the National Medical Security Administration, and 15 other departments. “Guiding Opinions on Further Improving and Implementing Active Birth Support Measures”, released on 25 July 2022. c. The General Office of the State Council. “Several Measures for Accelerating the Improvement of Birth Support Policy Systems and Promoting the Building of a Birth-friendly Society”, released on 19 October 2024. d. The General Office of the NHC, General Office of the Ministry of Education, and Office of the China Family Planning Association. “Action Plan for Promoting Reproductive Health (2023–2025)”, released on 24 July 2023. e. The General Office of the National Health Commission. “Core Information for Health Education on the Prevention and Treatment of Infertility”, released on 29 October 2021.

## Data Availability

These data are available from the first author.

## Abbreviations

The following abbreviations are used in this manuscript:

ART	Assisted reproductive technologies
ICPD	International Conference on Population Development
IVF	In vitro fertilization
IVF-ET	In Vitro Fertilization—Embryo Transfer
NHC	National Health Commission
NPO	Non-profit Organizations
SRHR	Sexual and Reproductive Health Rights
WHO	World Health Organization
